# A balanced score to predict survival of elderly patients newly diagnosed with glioblastoma

**DOI:** 10.1186/s13014-020-01549-9

**Published:** 2020-05-06

**Authors:** Christoph Straube, Kerstin A. Kessel, Stefanie Antoni, Jens Gempt, Bernhard Meyer, Juergen Schlegel, Friederike Schmidt-Graf, Stephanie E. Combs

**Affiliations:** 1 Department of Radiation Oncology, Klinikum rechts der Isar, Technical University of Munich, School of Medicine, Munich, Germany; 2 Deutsches Konsortium für translationale Krebsforschung (dktk), Partner Site Munich, Munich, Germany; 3 Department of Neuropathology, Technical University of Munich, Munich, Germany; 4 Department of Neurosurgery, Klinikum rechts der Isar, Technical University of Munich, Munich, Germany; 5 Department of Neurology, Klinikum rechts der Isar, Technical University of Munich, Munich, Germany; 6 Department of Radiation Sciences (DRS), Institut für Strahlenmedizin (IRM), Helmholtz Zentrum München (HMGU), Neuherberg, Germany

**Keywords:** Glioblastoma, Elderly, Score, Adjuvant treatment

## Abstract

**Background:**

Over the past years, several treatment regimens have been recommended for elderly patients with glioblastoma (GBM), ranging from ultrahypofractionated radiotherapy (RT) over monochemotherapy (ChT) to combined radiochemotherapy (RChT). The current guidelines recommend active treatment in elderly patients in cases with a KPS of at least 60%. We established a score for selecting patients with a very poor prognosis from patients with a better prognosis.

**Methods:**

One hundred eighty one patients ≥65 years old, histologically diagnosed with GBM, were retrospectively evaluated. Clinical characteristics were analysed for their impact on the overall survival (OS). Factors which were significant in univariate analysis (log-rank test, *p* < 0.05) were included in a multi-variate model (multi-variate Cox regression analysis, MVA). The 9-month OS for the significant factors after MVA (*p* < 0.05) was included in a prognostic score. Score sums with a median OS of < and > 6 months were summarized as Group A and B, respectively.

**Results:**

Age, KPS, MGMT status, the extent of resection, aphasia after surgery and motor dysfunction after surgery were significantly associated with OS on univariate analysis (*p* < 0.05). On MVA age (p 0.002), MGMT promotor methylation (p 0.013) and Karnofsky performance status (p 0.005) remained significant and were included in the score. Patients were divided into two groups, group A (median OS of 2.7 months) and group B (median OS of 7.8 months). The score was of prognostic significance, independent of the adjuvant treatment regimen.

**Conclusions:**

The score distinguishes patients with a poor prognosis from patients with a better prognosis. Its inclusion in future retrospective or prospective trials could help enhance the comparability of results. Before its employment on a routine basis, external validation is recommended.

## Introduction

Glioblastomas (GBM) are primary malignant brain tumours which are frequently present in the elderly cohort. The median age at upon diagnosis is about 64 years in several population-based studies [1–3]. Accordingly, a little less than 50% of GBM patients are older than 65 years upon diagnosis, and about one quarter is older than 72 years [1]. Within the past two decades, several treatment options for GBM have been evaluated. Currently, the international standard for adjuvant treatment after maximally safe resection is a combination of radiotherapy (RT) up to 60 Gy with concomitant and adjuvant chemotherapy (ChT) with temozolomide (TMZ) [4]. In cases with a hypermethylated O6–methylguanine DNA–methyl transferase-promotor (MGMT), a systemic treatment with lomustine (CCNU) and TMZ instead of TMZ alone can be considered as well [5]. Besides, the addition of tumour treatment fields (TTFields) has shown some activity in combination with adjuvant TMZ [6].

Age is a known risk factor for patients with GBM, the patient age being among the strongest single prognostic factor for the outcome [1–3]. The landmark trial from 2005 as well as the CeTeG trial included only patients up to an age of 70 years, with only 14% of patients older than 65 years in EORTC-NCIC 22981/26981 / CE.3 [4, 5]. The EA-14 trial had no upper threshold for age, yet the mean age in the trial was 55.8 years, while the detailed age distribution of the study population was not available. However, the median age of 55.8 years is also markedly below the median age of newly diagnosed GBM of the whole population of about 64 years [6, 7].

Based on an unplanned post hoc analysis of the EORTC-NCIC trial 22,981/26981 / CE.3, the efficacy of a combination treatment of elderly patients, defined by > 65 years, was questioned. The hazard ratios for a combination treatment worsened from 0.5 for patients younger than 50 years to 0.78 for patients older than 65 years. Notably, the patient number, especially in the oldest age group, was too small to draw a definitive conclusion [7]. RT, however, has shown its efficacy also in the aged cohort, where mono RT was tested against best supportive care (BSC) policy in patients at least 70 years of age with good performance status in a randomized trial. The trial resulted in an improvement of the overall survival (OS) due to RT, yet with a median OS of 6.7 months [8]. Subsequently, mono RT was tested against mono ChT in the NOA-08 trial, which showed an improvement of the OS in patients treated with TMZ only when the MGMT promotor was methylated. However, there was no difference in response to RT due to the MGMT promotor methylation [9, 10]. Notably, NOA-08 also included patients with anaplastic astrocytoma, while the vast majority were diagnosed for GBM [10]. Also, hypofractionated RT regimens were studied in the mono RT setting, which all showed similar efficacy in comparison to a mono RT with 60 Gy in 30 fractions [11–13]. Based on these results, a hypofractionated course of RT (40.05 Gy in 15 fractions) was tested against a combination of the hypofractionated RT with a concomitant and adjuvant TMZ-based ChT. The trial recruited patients at least 65 years of age with a good or fair performance status (Karnofsky Performance Status (KPS) ≥60%) and resulted in an improved OS with the combined treatment [14]. These results defined a new standard of care in elderly patients with GBM [15].

The heterogeneity of the treatment options and results in elderly GBM patients underline the importance of proper patient selection criteria. The current guidelines stratify the treatment recommendations for elderly patients (> 70 years) by the MGMT promotor methylation. Patients with a KPS < 70% are recommended for mono RT or mono ChT, depending on their MGMT promotor status [15]. Patients with a KPS of < 50% are generally not recommended for active treatment. These recommendations originate from the assumption of a poorer prognosis in this group, which is currently based only on discrete factors instead of an integrated score.

Prognostic scores for GBM were established as early as 1993 after results from the two RTOG trials were analysed in order to define prognostic groups by recursive partitioning analysis (RPA) [16]. The RPA retained its influence in the era of TMZ, which was shown by a re-evaluation of the RPA based on the EORTC-NCIC trial in 2006 [17]. Notably, both the RTOG studies (74–01 and 83–02) and the EORTC-NCIC trial included only patients up to an age of 70 years, and the RPA did not implement the impact of the MGMT promotor. Therefore, there is currently no prognostic score for elderly patients that includes the MGMT promoter status.

In contrast to primary GBM, scores are well established in recurrent disease [18]. Combs et al. were among the first to present a prognostic score for re-irradiation of recurrent glioma [19]. The score was externally validated and also improved [20–23], yet it was not validated for patients treated with subsequent bevacizumab [24]. Furthermore, a “re-irradiation risk score” (RRRS) was recently published and validated [25]. Similar to the Elderly-Score, all of these scores incorporated the age, but only the newer scores included the KPS. None of these scores included the MGMT methylation status, which is most likely by virtue of the fact that the scores were generated as re-irradiation scores [19, 21, 25, 26].

As the prognosis of a disease is important both for the recommendation of an adjuvant regimen in tumour boards and the counseling of patients, we aimed to generate a score based upon easily available factors which would be effectual towards identifying the group unlikely to benefit from any adjuvant treatment. To this end, we retrospectively analysed an elderly GBM cohort for prognostic factors, performed a univariate and multivariate analysis of prognostic factors, and ultimately generated a simple-to-use, balanced prognostic score.

## Methods

### Patients

The study included 181 patients at least 65 years of age who had undergone biopsy and/or resection of primary GBM from 01/2012 to 12/2016. Only patients with no prior diagnosis or treatment for an intracranial malignant disorder were eligible. The cases were extracted from the surgical logbook of the department of neurosurgery at the Klinikum rechts der Isar, Technical University of Munich, Germany, hence, all operated patients in the given time period were evaluated. The analysis was approved by the ethical board of our clinic (TUM vote 5625–12). The patient characteristics are summarized in Table 1. Furthermore, we calculated the molRPA as well as the elderly RPA for every patient (supplemental Table 1) [27, 28].

**Table 1:** patient characteristics

Characteristics	Total	Percent
**Gender**		
Male	97	53.6%
Female	84	46.4%
**Age**	74.2 (65–90)	
65–69.9	50	27.6%
70–74.9	42	23.2%
75–79.9	49	27.1%
> 80	40	22.1%
**KPS**	80 (20–100)	
90–100	52	28.7%
70–80	76	42.0%
50–60	43	23.8%
20–40	10	5.5%
**MGMT**		
MGMT methylated	38	21.0%
MGMT unmethylated	63	34.8%
MGMT unknown	80	44.2%
**IDH**		
IDH wildtype	111	61.3%
IDH unknown	70	38.7%
**EOR**		
Biopsy	33	18.2%
STR	89	49.2%
GTR	59	32.6%
**Seizures**		
no Seizures	145	80.1%
focal Seizures	12	6.6%
generalized Seizures	22	12.2%
Epileptic status	1	0.6%
unknown	1	0.6%
**Motor deficit before Surgery**		
no motor deficit	105	58.0%
any motor deficit	76	42.0%
**Motor deficit after Surgery**		
no motor deficit	89	49.2%
any motor deficit	92	50.8%
**Aphasia before Surgery**		
no aphasia	129	71.3%
any aphasia	52	28.7%
**Aphasia after Surgery**		
no aphasia	107	78.7%
any aphasia	29	21.3%
unknown	45	n/a
**Treatment after Surgery**		
BSC	47	26.0%
RT	72	39.8%
ChT	7	3.9%
RChT	55	30.4%

Abbreviations: *KPS* Karnofsky Performance Scale, *MGMT* O6-Methylguanine DNA-methyl transferase, *IDH* Isocitrate dehydrogenase, *EOR* Extent of resection, *STR* Subtotal resection, *GTR* Gross total resection, *BSC* Best supportive care, *RT* Radiotherapy, *ChT* Chemotherapy, *RChT* Radio-chemotherapy

The clinical records of all cases were reviewed retrospectively for pathological findings (i.e., histology, IDH mutation status, MGMT promotor hypermethylation), symptoms prior to surgery (focal seizures, motor deficits), postoperative Karnofsky performance status (KPS), the extent of resection (EOR) as well as the use of adjuvant treatments and the date of death or last contact.

At our centre, all treatment decisions are discussed and approved by a multidisciplinary tumour board. Patients are included in a narrow follow-up program with clinical visits and contrast-enhanced MRI at least every 3 months.

### Treatments

#### Radiation therapy

Radiotherapy was performed either as mono RT (RT) or in combination with temozolomide (RChT). Fractionation was either standard fractionated with single doses from 1.8 to 2.0 Gy to a total dose of 50.4 to 60.0 Gy, depending on the location of the disease, or hypofractionated with 2.5 to 3.0 Gy per fraction to a total dose of 35 to 45 Gy. In RChT, only single fractions of 1.8 to 2.0 to a total dose of 60 Gy (median, range 50.4 to 60 Gy) were used. Hypofractionated RChT, as proposed by Perry et al., was implemented after the publication of the results in 2017. Hence, hypofractionated RChT was not included in this cohort [14].

#### Chemotherapy

When chemotherapy was used concomitantly (RChT), then temozolomide (TMZ) was used in a dose of 75 mg/m^2^. Adjuvant to RChT or as single agent treatment without RT (ChT), TMZ was prescribed in the 5/28 regimen. Hence, 150 to 200 mg/m^2^/d were given on five consecutive days with a total cycle length of 28 days. This regimen is in line with the Stupp Regimen [4].

#### Best supportive care

If a best supportive care (BSC) policy was initiated, a specialized palliative care team was involved. BSC usually includes a sufficient medical anticonvulsive treatment, psychological guidance as well as administration of corticosteroids if necessary. Follow-up imaging is generally not excluded, but the frequency thereof is usually lower.

#### Salvage strategies

Forty-three patients underwent subsequent lines of therapy, such as re-irradiation, re-chemotherapy, a second surgery or a combination of these. Surgery was the most prevalent rescue strategy, with 26 patients undergoing a second surgery for progressive disease. In addition, 26 patients received some kind of ChT as part of their salvage treatment. Thirteen patients received a second course of radiotherapy. Furthermore, eleven patients underwent a second, and three patients a third salvage treatment. As the utilization of salvage treatments is not predictable when the initial diagnosis is made, we did not include any data on salvage treatments in the model and the score, respectively.

### Statistical analysis and score generation

The statistical analysis was performed with SPSS v18 (IBM). The influence of single variables was compared with the Kaplan Meier method, and a *p*-value of < 0.05 in the log rank test was considered significant (univariate analysis, UVA). The Kaplan Meier analysis had two purposes: first, to select variables for multi-variate analysis, and second, to quantify the strength of each variable. Only factors that were known prior to adjuvant treatment were selected. In order to exclude dependent variables, the correlation between significant factors from UVA was analysed with the Chi-Square test.

Variables, which gained significance on UVA, were included in the multi-variate Cox regression analysis (MVA). The MVA was carried out in order to exclude variables with only a minor influence. A *p*-value of < 0.05 was considered significant.

To generate a prognostic score, variables were included which were independent predictors for the OS and had a significant impact on the MVA model. For each of the selected variables, the actuarial 9 month overall survival (OS) rate was divided by 10 and equaled the subscore for the variable. Thus, the resulting subscore represents the influence of the parameter. The subscores of all significant parameters were totalled.

To generate a threshold, we reviewed the Kaplan Meier curves for every total score separately. A median OS of < 6 months would result in a high proportion of patients dying before the end of RChT followed by six cycles of adjuvant ChT. Hence, these scores were arbitrarily defined as “Group A”. Vice versa, total scores with an OS of at least 6 months from diagnosis were included in “Group B”. This method was previously used in scores from Rades et al. [29].

In order to test whether the score had prognostic value independent of the adjuvant treatment, we performed a multi-variate Cox regression analysis in regard to the score as well as the kind of adjuvant treatment, the latter presumably being a confounder of the native prognosis. Also, here a *p*-value < 0.05 was considered significant.

## Results

On UVA, the age upon diagnosis (*p* < 0.001), the MGMT promotor methylation status (p 0.035), the KPS (*p* < 0.001), the EOR (p 0.004), the presence of motor deficits after surgery (p 0.001) as well as the presence of aphasia after surgery (p 0.044) were significantly associated with the OS. Neither gender, the presence of seizures upon diagnosis nor motor deficits nor aphasia before surgery were significant parameters (Table 2). Age was correlated with a decreased rating in the KPS (Chi-Square, p 0.002) as well as with the EOR (p 0.009), while the KPS was also related to the presence of motor deficits (*p* < 0.0001) (Table 3).

**Table 2:** UVA of the investigated parameters

UVA	3 m OS	6 m OS	9 m OS	12 m OS	*P*-value	n	mOS
**Age**					**< 0.001**		
65–69.9	88%	67%	45%	43%		50	7.8
70–74.9	73%	56%	44%	32%		42	6.6
75–79.9	71%	47%	27%	13%		49	5.5
> 80 years	52%	22%	10%	2%		40	3.2
**Gender**					0.806		
Female	72%	49%	35%	21%		75	6.0
Male	73%	46%	29%	23%		86	5.4
**MGMT**					**0.035**		
unknown or non-methylated	69%	42%	28%	20%		143	5.0
methylated	83%	72%	48%	34%		38	8.7
**KPS**					**< 0.001**		
10–40%	29%	0%	0%	0%		7	2.4
50–60%	60%	25%	15%	12%		40	3.4
70–80%	76%	54%	37%	25%		68	6.4
90–100%	80%	65%	43%	32%		46	8.1
**EOR**					**0.004**		
Biopsy	57%	30%	13%	6%		30	3.1
STR	74%	50%	32%	22%		89	5.9
GTR	76%	55%	39%	31%		59	6.4
**Seizures**					0.264		
no seizures	71%	45%	29%	21%		141	5.6
generalized	68%	47%	32%	16%		22	6.2
focal	82%	73%	54%	45%		12	13.5
**Motor deficits before surgery**					0.073		
no motor deficit	72%	55%	36%	27%		105	6.4
any motor deficit	74%	37%	25%	17%		76	4.9
**Motor deficits after surgery**					**0.001**		
no motor deficit	82%	65%	45%	35%		89	7.8
any motor deficit	63%	31%	17%	11%		92	4.1
**Aphasia before surgery**					0.132		
no aphasia	72%	52%	35%	27%		129	6.2
aphasia	74%	38%	23%	13%		52	5.0
**Aphasia after surgery**					**0.044**		
no aphasia	85%	62%	42%	31%		107	7.3
aphasia	81%	44%	29%	14%		29	5.7

Abbreviations: *UVA* Univariate analysis, *KPS* Karnofsky Performance Scale, *MGMT* O6-Methylguanine DNA-methyl transferase, *EOR* Extent of resection, *STR* Subtotal resection, *GTR* Gross total resection

**Table 3:** Chi-Square test for parameters which were significant after UVA

	Age	KPS	MGMT	EOR	Motor Deficit	Aphasia
**Age**		**0.002**	0.119	**0.009**	0.176	0.73
**KPS**	**0.002**		0.498	0.236	**< 0.001**	0.619
**MGMT**	0.119	0.498		0.791	0.427	0.337
**EOR**	**0.009**	0.236	0.791		0.265	0.337
**Motor Deficit**	0.176	**< 0.001**	0.427	0.265		0.412
**Aphasia**	0.73	0.619	0.994	0.337	0.412	

Abbreviations: *KPS* Karnofsky Performance Scale, *MGMT* O6-Methylguanine DNA-methyl transferase, *EOR* Extent of resection

**Fig. 1:**
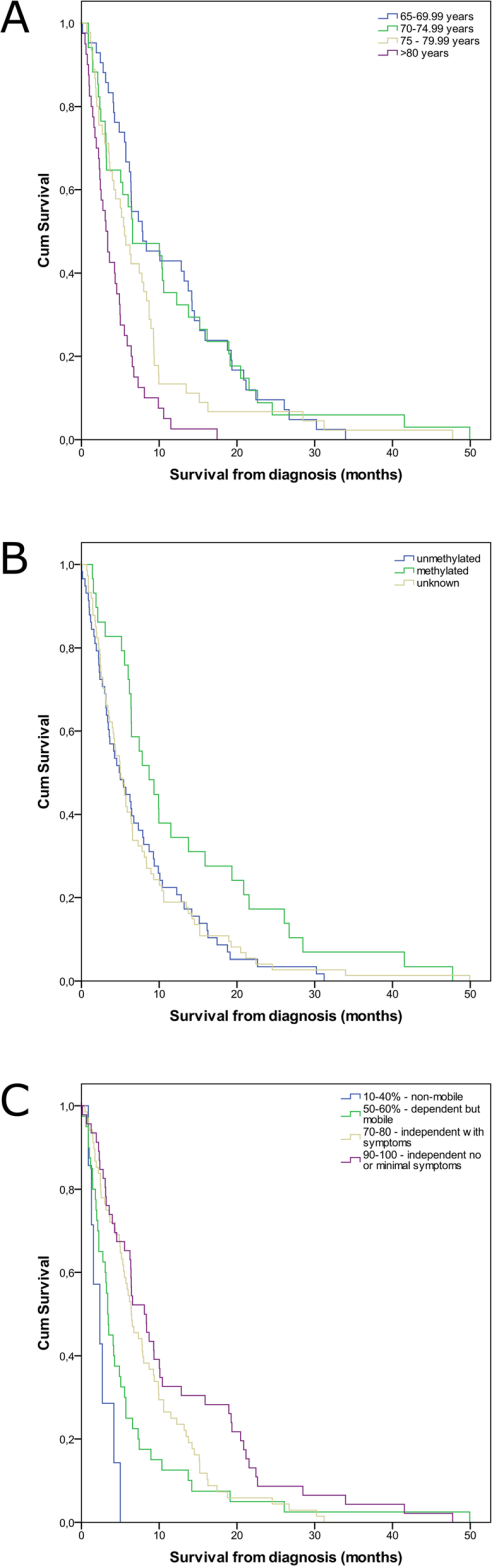
Kaplan Meier curves for the factors involved in the score. **a**) Age **b**) MGMT C) KPS

**Fig. 2:**
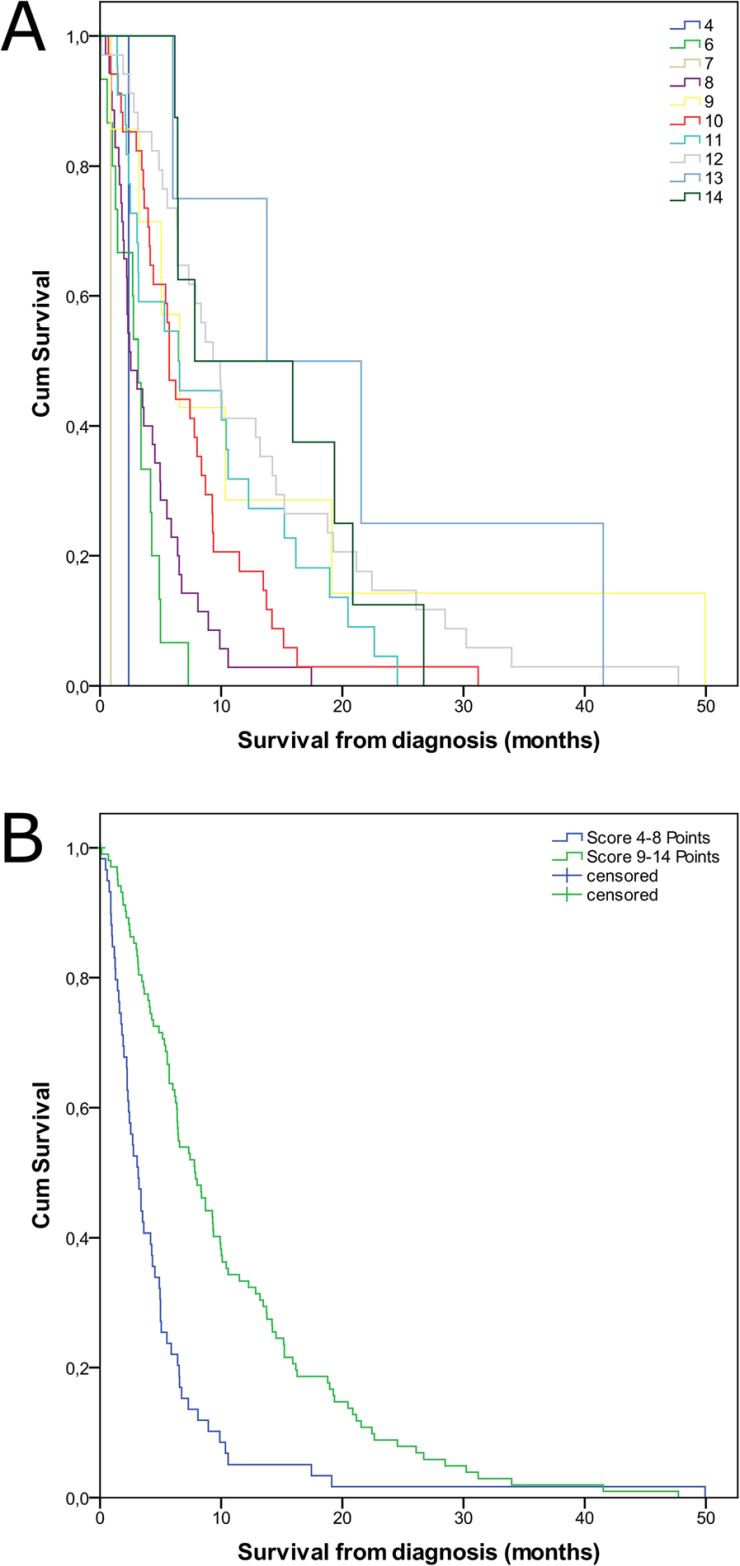
Kaplan Meier curves for the Elderly-Score, **a**) for all Scores and **b**) with a threshold of 8

**Fig. 3:**
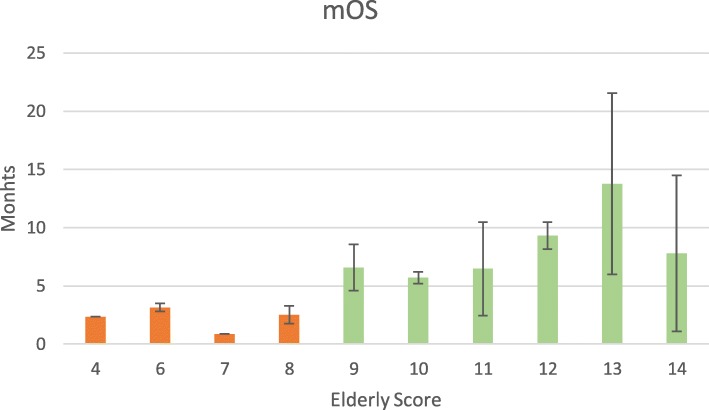
Scoring points and mOS of individual patients. The error bars represent the standard error of the mOS distribution

On MVA, only age (p 0.002), MGMT promotor methylation (p 0.013) and KPS (p 0.005) remained significant (Table 4). The Kaplan-Meier curves of these parameters are shown in Fig. 1. The significant parameters were included in Table 5, Fig. 2 A and 3. Patients with more than eight scoring points had a mOS of more than 6 months, patients with four to eight points had a mOS of fewer than 6 months (Fig. 2b and Fig. 3, supplemental Table 2). Therefore, we categorized patients with 4–8 points as Group A and patients with > 8 points as Group B (Fig. 2b, supplemental Table 2). The two groups significantly differed in mOS (mOS 2.7 vs 7.8 months, log rank test: *p* < 0.001, Fig. 2b, supplemental Table 2).

**Table 4:** – MVA of the parameters which were significant in UVA

MVA	HR	95% CI	***P***-value
**Age**			
65–69.9	Reference		**0.002**
70–74.9	1.179	0.704–1.972	0.532
75–79.9	1.53	0.892–2.626	0.123
> 80 years	2.878	1.651–5.017	< 0.001
**MGMT**			
methylated	Reference		
unknown or non-methylated	1.91	1.147–3.180	**0.013**
**KPS**			
90–100%	Reference		**0.005**
70–80%	0.85	0.534–1.351	0.491
50–60%	1.949	1.104–3.439	0.021
10–40%	4.433	0.92–21.352	0.063
**EOR**			
GTR	Reference		0.275
STR	1.331	0.855–2.072	0.205
Biopsy	1.567	0.868–2.830	0.136
**Motor deficits after surgery**			
no motor deficit	Reference		
any motor deficit	1.117	0.727–1.715	0.615
**Aphasia after surgery**			
no aphasia	Reference		
aphasia	1.466	0.907–2.370	0.118

Abbreviations: *MVA* Multivariate analysis, *HR* Hazard ratio, *CI* Confidence interval, *KPS* Karnofsky Performance Scale, *MGMT* O6-Methylguanine DNA-methyl transferase, *EOR* Extent of resection, *STR* Subtotal resection, *GTR* Gross total resection

**Table 5:** Scoring points for the Elderly-Score

Item	Score
**Age**	
65–69.9	5
70–74.9	4
75–79.9	3
> 80 years	1
**KPS**	
10–40%	0
50–60%	2
70–100%	4
**MGMT**	
unknown or non- methylated	3
methylated	5

Abbreviations: *KPS* Karnofsky Performance Scale, *MGMT* O6-Methylguanine DNA-methyl transferase

**Fig. 4:**
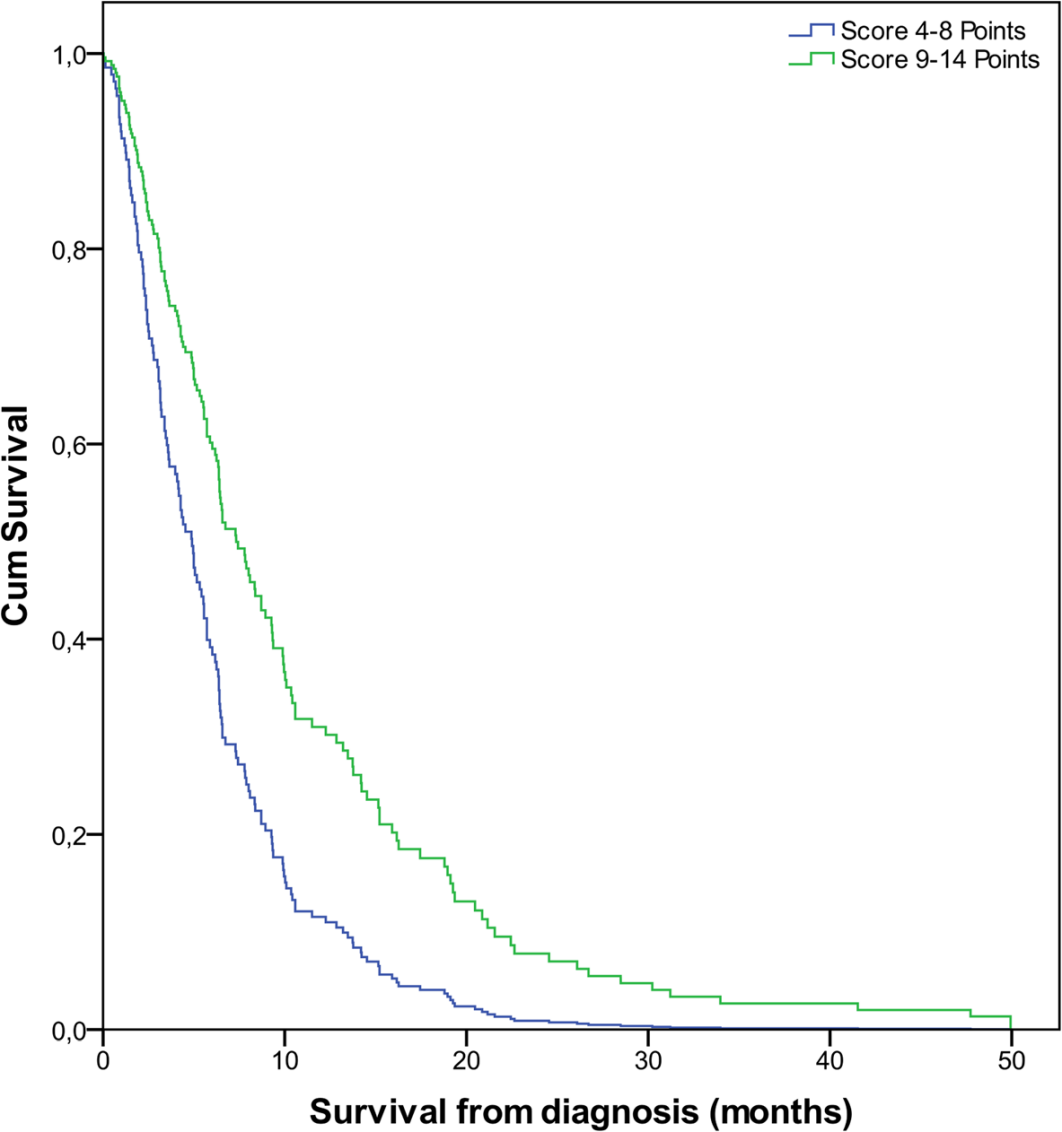
Cox Regression Analysis of the Elderly-Score controlled for the first treatment regimen (*p*<0.001). A threshold of 8 points was chosen, as a score of at least 9 points resulted in a median OS of > 6 months, while a lower score reached survival times of <6 months

The generated score was a predictor for OS independent of the subsequent adjuvant regimen. This was proven by a second MVA, including the prognostic group A vs. group B as well as the any active adjuvant treatment regimen vs. BSC (Fig. 4). Both the treatment regimen (*p* < 0.001) and the groups (*p* < 0.001) were significant in this model.

## Discussion

We generated a prognostic score for elderly patients with newly diagnosed GBM. The score is based on the KPS after surgery, the age of the patients as well as the MGMT promotor methylation and balances these factors one against the others. With the score, prognostic groups were discriminated, with group A resulting in a poor median OS of 2.8 months as compared to group B with a median OS of 7.9 months. The score is deemed to be prognostic, independent of the subsequent adjuvant treatment regimen and can select a patient group, which is unlikely to benefit from adjuvant treatment. As generally recommended for patients with a life expectancy of less than 3 months, an advanced palliative care plan should be initiated for patients in group A [30].

Interestingly, the EOR did not reach significance in MVA, despite its high significance in UVA. One reason for this could be that the EOR was only trichotomized for biopsy, STR, and GTR. Possibly, volumetric analysis of the remaining tumour mass could add further information [31, 32], yet a sophisticated volumetric analysis of the remaining tumour is beyond the scope of this manuscript. Another reason could be the statistical importance of the postoperative performance status as well as the MGMT promotor methylation in our model. These factors might be of more importance in the elderly cohort and therefore, potentially diminish the benefit of an increased EOR. Notably, the EOR is an established prognostic factor in GBM, but the studies supporting this factor were conducted with predominantly young patients [33]. Especially in the more vulnerable elderly cohort, the EOR needs, therefore, to be balanced against potential deficits caused by resection or its possible complications. In support of this hypothesis, Karsy et al. described a better OS after gross total resection only in patients with no additional deficits after surgery [34]. Also, in contrast to our findings, the Elderly-RPA from Scott et al. used the EOR dichotomized by subtotal and gross total resection vs biopsy as the first node in their RPA-based decision tree [28]. However, the Elderly-RPA did not include the MGMT promotor methylation, a prognostic marker that has already been shown to be prognostic as well as predictive in prospective trials, also in elderly patients [9, 14, 35]. Concerning the EOR, it is necessary to clarify that the data analysis was performed in order to generate a score to enhance decision-making after surgery, not to analyse the impact of the EOR for the prognosis of the patient or to guide decisions for the planned approach or EOR.

The initially proposed RPA stratified six prognostic groups that were stratified by ages of more or less than 50 years in the first instance. Further stratifications included the histology, the KPS as well as several other factors [16]. The RPA was renewed and simplified after the publication of the EORTC-NCIC trial in 2006. Hence, the adapted EORTC-RPA focused on GBM and stratified by the age (+/− 50 years), the EOR, the performance status (WHO +/− 0) and alterations in the mini-mental status examination (MMSE) [17]. Notably the early, as well as the adapted EORTC-RPA, was based on clinical trials, which excluded patients older than 70. Therefore, while still a valuable tool, the initial RPA offers only limited help when elderly patients with GBM are counseled. Several other RPAs have been generated since then, one of the more recent is the GBM-molRPA presented and validated by Woo et al. and the elderly-RPA presented by Scott et al. [27, 28, 36]. The molRPA stratifies by the age of 50 years, yet only MGMT negative patients are dichotomized by age. Consequently, only seven patients in our cohort received a molRPA I (median OS 21.5 months, MGMT methylated, GTR, KPS at least 90%) and 76 patients a molRPA II with a median OS of only slightly above 6 months (6.4 months, supplemental Table 1). In comparison, elderly-RPA I patients gained a median OS of 8.3 months in our cohort (GTR or STR, < 75.5 years; *p* < 0.001, supplemental Table 1) [28]. The patients in class II to IV gained a median OS of clearly below 6 months (4.9 months, 3.2 months, 3.1 months; *p* < 0.001, supplemental Table 1). Stratification by the elderly-RPA as well as by the molRPA, therefore, selected a different cohort as best prognosis group, as only 84 patients were classified as Elderly-RPA I, while 127 patients in our cohort gained an Elderly-Score of nine or higher which was associated with a median OS of 7.9 months. This finding can be explained by one of the major disadvantages of all RPAs, as continuous variables, such as age, can only be included as dichotomized values. Furthermore, RPAs tend to over-fit the data, especially when a multitude of factors is included. When compared to a decision tree, scores offer the advantageous option of a positive prognostic value compensating for a negative one. For example, in order to achieve a score of > 8, a 76-year-old patient with a KPS of 50–60% can still reach a score of 10 when the MGMT promotor is methylated. A simplified score, such as the Elderly-Score presented in this manuscript, therefore might be more feasible to guide therapy decisions in daily practice.

Besides score practicality, some limitations should be noted. Firstly, the present work is based on retrospective data. While a selection bias was avoided by focusing on a distinct timeframe as well as by including all patients with a GBM histology during this period, the data were not collected in accordance to a trial protocol, but to the standard operation procedures of our hospital. This also resulted in missing data, especially since the MGMT promotor methylation status was not available in about 44% of patients. Of note, the MGMT status was not a standard criterion until 2016-WHO classification [37]. In the elderly cohort, however, an unknown MGMT status can be equated to a negative MGMT status (Fig. 1b), especially when patients are predominantly treated with mono RT (Table 1). The results from NOA-08, which showed no prognostic relevance of the MGMT methylation status in patients treated with mono RT, substantiate this hypothesis [10]. When patients were treated with hypofractionated RChT as in EORTC 26062–22,061 then the subgroup of MGMT non-methylated patients showed only a borderline significant improvement of the OS in patients treated with hypofractionated RChT as compared to hypofractionated RT [14].

## Conclusion

We presented a simple score for prognostication of elderly patients with newly diagnosed GBM. The score is based on only three factors that are available directly after histological diagnosis, thus easy to use. Validation of the score by an independent cohort is recommended. The score could be used to enhance the comparability of reported results if included in future publications. To date, it may be used to support treatment decisions.

## Supplementary information

**Additional file 1: Table S1.** The molRPA [27], as well as the Elderly RPA [28], were calculated for all patients. The median OS was calculated with the Kaplan-Meier-Method. A log rank test was done to compare the OS in the RPA-groups I-III and I-IV. A *p*-value of < 0.05 was considered significant.

**Additional file 2: Table S2.** Patients at Risk in Kaplan Meier Curves.

## Data Availability

The dataset supporting the conclusions of this article contains clinical as well as demographic data. Therefore, sharing of the entire dataset online was restricted by the local ethical committee. However, selected data can be requested from the corresponding author.
