# Supplementary material for: A balanced score to predict survival of elderly patients newly diagnosed with glioblastoma
Source: Radiat Oncol. 2020 May 6;15:97. doi: 10.1186/s13014-020-01549-9 (PMC7201994; doi:10.1186/s13014-020-01549-9)
Supplement: Supplementary file 1 — Additional file 1: Table S1. The molRPA [27], as well as the Elderly RPA [28], were calculated for all patients. The median OS was calculated with the Kaplan-Meier-Method. A log rank test was done to compare the OS in the RPA-groups I-III and I-IV. A p-value of < 0.05 was considered significant. [file 13014_2020_1549_MOESM1_ESM.docx]

**Supplemental Table 1:** The molRPA [27], as well as the Elderly RPA [28], were calculated for all patients. The median OS was calculated with the Kaplan-Meier-Method. A log rank test was done to compare the OS in the RPA-groups I-III and I-IV. A p-value of <0.05 was considered significant.

| **molRPA** | **n** | **OS** | **p (Log-Rank)** |
| --- | --- | --- | --- |
| **I** | 7 | 21.5 | <0.001 |
| **II** | 76 | 6.4 |  |
| **III** | 98 | 4.4 |  |
|  |  |  |  |
| **Elderly-RPA** | **n** | **OS** | **p (Log-Rank)** |
| **I** | 84 | 8.3 | <0.001 |
| **II** | 64 | 4.9 |  |
| **III** | 20 | 3.2 |  |
| **IV** | 13 | 3.1 |  |
