# Supplementary material for: A balanced score to predict survival of elderly patients newly diagnosed with glioblastoma
Source: Radiat Oncol. 2020 May 6;15:97. doi: 10.1186/s13014-020-01549-9 (PMC7201994; doi:10.1186/s13014-020-01549-9)
Supplement: Supplementary file 2 — Additional file 2: Table S2. Patients at Risk in Kaplan Meier Curves. [file 13014_2020_1549_MOESM2_ESM.docx]

| **Supplemental Table 2: Patients at Risk in Kaplan Meier Curves** | | | | | | | |
| --- | --- | --- | --- | --- | --- | --- | --- |
|  |  |  |  |  |  |  |  |
| **Figure 1a** |  |  |  |  |  |  |  |
| **Age per 5 years** | **Total** | **6 months** | **12 months** | **18 months** | **24 months** | **30 months** | **36 months** |
| **65-69.99 years** | 50 | 28 | 18 | 10 | 4 | 2 | 0 |
| **70-74.99 years** | 42 | 20 | 12 | 8 | 3 | 2 | 2 |
| **75-79.99 years** | 49 | 21 | 6 | 3 | 3 | 2 | 1 |
| **>80 years** | 40 | 9 | 1 | 0 | 0 | 0 | 0 |
|  |  |  |  |  |  |  |  |
| **Figure 1b** |  |  |  |  |  |  |  |
| **MGMT promotor methylation** | **Total** | **6 months** | **12 months** | **18 months** | **24 months** | **30 months** | **36 months** |
| **not methylated** | 63 | 26 | 13 | 5 | 2 | 2 | 0 |
| **methylated** | 38 | 22 | 10 | 8 | 5 | 2 | 0 |
| **unknown** | 80 | 30 | 14 | 8 | 3 | 2 | 1 |
|  |  |  |  |  |  |  |  |
| **Figure 1c** |  |  |  |  |  |  |  |
| **Karnofsky Performance Scale** | **Total** | **6 months** | **12 months** | **18 months** | **24 months** | **30 months** | **36 months** |
| **10-40%** | 7 | 0 | 0 | 0 | 0 | 0 | 0 |
| **50-60%** | 43 | 10 | 5 | 3 | 2 | 1 | 1 |
| **70-80%** | 76 | 38 | 17 | 5 | 4 | 2 | 0 |
| **90-100%** | 52 | 30 | 15 | 13 | 4 | 3 | 2 |
|  |  |  |  |  |  |  |  |
| **Figure 2a** |  |  |  |  |  |  |  |
| **Total score** | **Total** | **6 months** | **12 months** | **18 months** | **24 months** | **30 months** | **36 months** |
| **4** | 1 | 0 | 0 | 0 | 0 | 0 | 0 |
| **6** | 17 | 1 | 0 | 0 | 0 | 0 | 0 |
| **7** | 1 | 0 | 0 | 0 | 0 | 0 | 0 |
| **8** | 35 | 8 | 1 | 0 | 0 | 0 | 0 |
| **9** | 10 | 4 | 2 | 2 | 1 | 1 | 1 |
| **10** | 36 | 16 | 6 | 1 | 1 | 1 | 0 |
| **11** | 24 | 12 | 7 | 4 | 1 | 0 | 0 |
| **12** | 38 | 25 | 14 | 9 | 5 | 3 | 1 |
| **13** | 7 | 4 | 3 | 2 | 1 | 1 | 1 |
| **14** | 12 | 8 | 4 | 3 | 1 | 0 | 0 |
|  |  |  |  |  |  |  |  |
| **Figure 2b** |  |  |  |  |  |  |  |
| **Categories** | **Total** | **6 months** | **12 months** | **18 months** | **24 months** | **30 months** | **36 months** |
| **4-8 points** | 54 | 9 | 1 | 0 | 0 | 0 | 0 |
| **9-14 points** | 127 | 69 | 36 | 21 | 10 | 6 | 3 |
